# Supplementary material for: A particle-field approach bridges phase separation and collective motion in active matter
Source: Nat Commun. 2020 Oct 23;11:5365. doi: 10.1038/s41467-020-18978-5 (PMC7584633; doi:10.1038/s41467-020-18978-5)
Supplement: Supplementary file 1 — Supplementary Information [file 41467_2020_18978_MOESM1_ESM.pdf]

SUPPLEMENTARY INFORMATION

A particle-field approach bridges phase separation and collective motion in active matter

Robert Großmann, Igor S. Aranson, and Fernando Peruani

SUPPLEMENTARY NOTE 1: BINARY INTERACTION BY OVERLAP MINIMIZATION

Smooth particle representation and overlap calculation. A single rod at position $\mathbf{r}_k$ with orientation φ_k is considered to be an elliptical object which is represented by a smooth field $\psi(\mathbf{r};\mathbf{r}_k,\varphi_k)$. This field can be rewritten as

$$\psi(\mathbf{r};\mathbf{r}_k,\varphi_k) = e^{-(\mathbf{r}-\mathbf{r}_k)\cdot\left[\frac{\mathbb{1}+\mathcal{Q}[\varphi_k]}{4l_\parallel^2}+\frac{\mathbb{1}-\mathcal{Q}[\varphi_k]}{4l_\perp^2}\right]\cdot(\mathbf{r}-\mathbf{r}_k)} \tag{1}$$

using the matrix

$$\mathcal{Q}[\varphi] = \begin{pmatrix} \cos(2\varphi) & \sin(2\varphi) \\ \sin(2\varphi) & -\cos(2\varphi) \end{pmatrix} \tag{2}$$

that encodes the orientation of a rod in two dimensions. Its eigenvectors $\mathbf{e}_\parallel[\varphi]=(\cos\varphi,\sin\varphi)^T$ and $\mathbf{e}_\perp[\varphi]=(-\sin\varphi,\cos\varphi)^T$ determine the major and minor axis, respectively, as a function of the polar angle φ. As in the main text, the dimensions of the major and minor axis are parametrized by $l_\parallel$ and $l_\perp$.

The Gaussianity of the field ψ facilitates the analytical calculation of the overlap

$$\mathcal{I}(\mathbf{r}_k,\mathbf{r}_j;\varphi_k,\varphi_j) = \int d^2r\,\psi(\mathbf{r};\mathbf{r}_k,\varphi_k)\,\psi(\mathbf{r};\mathbf{r}_j,\varphi_j) \tag{3}$$

of two rods. Due to the convolution structure of this integral, it may be solved via Fourier transformation methods yielding

$$\mathcal{I} = \mathcal{I}_0\, e^{-\frac{1}{1-\varepsilon^2\cos^2(\varphi_k-\varphi_j)}\cdot\frac{(\mathbf{r}_k-\mathbf{r}_j)\cdot[\mathbb{1}-\varepsilon\bar{\mathcal{Q}}(\varphi_k,\varphi_j)]\cdot(\mathbf{r}_k-\mathbf{r}_j)}{2\left(l_\parallel^2+l_\perp^2\right)}} \tag{4}$$

with the position-independent prefactor

$$\mathcal{I}_0 = \mathcal{I}_0(\varphi_k,\varphi_j) = \pi l_\parallel l_\perp \sqrt{\frac{1-\varepsilon^2}{1-\varepsilon^2\cos^2(\varphi_k-\varphi_j)}} \tag{5}$$

which reflects the overlap of two rods whose centers of mass are identical. The mean nematic matrix $\bar{\mathcal{Q}}(\varphi_k,\varphi_j) = \frac{1}{2}\big(\mathcal{Q}[\varphi_k]+\mathcal{Q}[\varphi_j]\big)$ in the exponent of Supplementary Eq. (4) accounts for the spatial anisotropy in dependence of the relative rod orientation.

Interaction energy. The central question from the modeling point of view is how to derive the interaction from the overlap $\mathcal{I}$. The basic idea is that spatially extended objects should repel if they come close to each other due to steric effects. We recall that the interaction energy was assumed to be binary, i.e. the total interaction energy is the sum of binary contributions:

$$\mathcal{U} = \frac{1}{2}\sum_{k,j}^{N} u_2(\mathbf{r}_k-\mathbf{r}_j;\varphi_k,\varphi_j). \tag{6}$$

The interaction energy u_2 should be an increasing function of the overlap $\mathcal{I}$. A natural assumption could therefore be the direct proportionality of the overlap to the interaction energy: $u_2 \propto \mathcal{I}$. Accordingly, pairs of particles which are far apart do not contribute to the interaction energy, whereas there is an energy cost associated to their mutual overlap as they approach each other. This model works well as long as overlaps remain small. However, spurious torques emerge in this model if large overlaps can occur, e.g. at very high particle densities or for high activity. To illustrate this issue, let us consider two rods whose centers of mass are close-by (Supplementary Fig. 1); in particular, we compare the overlap for two particles whose body axis are aligned with a configuration where their orientations form a cross. It is self-evident that the cross configuration would correspond to the configuration of lower potential energy as the overlap is smaller than in the aligned situation. Physically, it is, however, questionable why two rods which slide past each other should align in a perpendicular fashion. Therefore, we argue that the ansatz $u_2 \propto \mathcal{I}$ is questionable for driven nonequilibrium systems if significant overlaps are likely to occur. At the end of this section, we give the precise mathematical expression for these spurious torques for completeness.

In order to avoid the emergence of spurious torques for close-by particles, we rethink the problem again. According to the Buckingham π theorem [1], the most general dependence of the binary interaction energy on the overlap is $u_2 = \kappa\mathcal{F}[\xi]$,

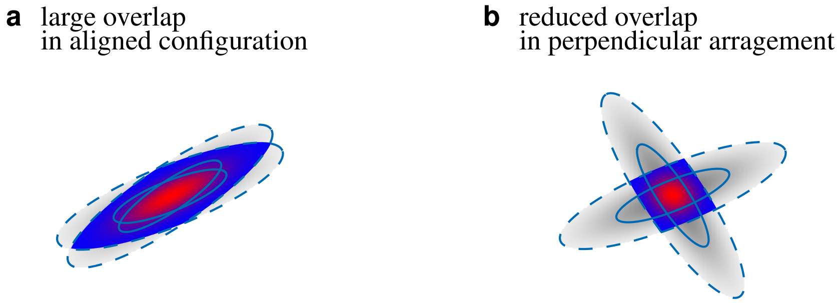

Supplementary Figure 1 | Sketch of two rods whose centers of mass are close-by. a The overlap (shown in color) of two nematically aligned rods is large. In contrast, the overlap is minimized by perpendicular alignment of the long body axis as illustrated in panel **b**. Therefore, spurious torques favoring perpendicular alignment would be implied if the interaction energy was chosen directly proportional to the overlap itself as this configuration corresponds to a state of minimal energy.

where κ is a constant with the units of energy and $\mathcal{F}[\xi]$ is a nonlinear function of the dimensionless variable $\xi = \mathcal{I}/a_0$, where $\mathcal{I}$ is the overlap and a_0 is a characteristic surface. We often use $\mathcal{F}[\xi] = \xi^\gamma$, with $\gamma > 0$ since we expect u_2 to be an increasing function of the overlap. Other functional forms may be used without affecting the observed large-scale behavior of the system qualitatively. It is also possible to implement a dependence $\mathcal{F}[\xi]$ that diverges for $\xi > \xi_*$ to ensure a maximal compressibility of the particles. Moreover, we want Onsager's argument [2] to be valid for a system of elongated passive particles represented by the proposed phase field, i.e. we expect the particles to get nematically aligned at the mean-field level locally. It turns out that the choice $a_0 = \mathcal{I}_0$ is consistent with Onsager's argument. In summary, the interaction energy u_2 is expressed as

$$u_2(\mathbf{r}_k-\mathbf{r}_j;\varphi_k,\varphi_j)=\kappa\mathcal{F}\left[\frac{\mathcal{I}(\mathbf{r}_k,\mathbf{r}_j;\varphi_k,\varphi_j)}{\mathcal{I}_0(\varphi_k,\varphi_j)}\right]=\kappa\mathcal{F}\left[e^{-\frac{(\mathbf{r}_k-\mathbf{r}_j)\cdot[\mathbb{1}-\varepsilon\bar{\mathcal{Q}}(\varphi_k,\varphi_j)]\cdot(\mathbf{r}_k-\mathbf{r}_j)}{2[1-\varepsilon^2\cos^2(\varphi_k-\varphi_j)]\left(l_\parallel^2+l_\perp^2\right)}}\right]. \tag{7}$$

Accordingly, the binary interaction energy reduces to a constant if two particles are close by ($\mathbf{r}_j \simeq \mathbf{r}_k$) and, thus, the emergence of spurious torques is excluded.

Derivation of force and torque. In order to derive the force and torque, the interaction energy should be differentiated with respect to the position and orientation of a focal particle: $\mathbf{F}_k = -\nabla_k \mathcal{U}$ and $M_k = -\partial_{\varphi_k}\mathcal{U}$. As the interaction is binary by construction, we can write force and torque as a sum via

$$\mathbf{F}_k = -\nabla_k \mathcal{U} = \textstyle\sum_{j=1;j\neq k}^{N} \mathbf{f}_2(\mathbf{r}_k - \mathbf{r}_j, \varphi_k, \varphi_j), \tag{8a}$$

$$M_k = -\partial_{\varphi_k}\mathcal{U} = \textstyle\sum_{j=1;j\neq k}^{N} m_2(\mathbf{r}_k - \mathbf{r}_j, \varphi_k, \varphi_j), \tag{8b}$$

where

$$\mathbf{f}_2(\mathbf{r},\varphi,\varphi') = -\kappa\nabla_{\mathbf{r}}\mathcal{F}\left[e^{-\frac{\mathbf{r}\cdot[\mathbb{1}-\varepsilon\bar{\mathcal{Q}}(\varphi,\varphi')]\cdot\mathbf{r}}{2[1-\varepsilon^2\cos^2(\varphi-\varphi')]\left(l_\parallel^2+l_\perp^2\right)}}\right], \tag{9a}$$

$$m_2(\mathbf{r},\varphi,\varphi') = -\kappa\partial_{\varphi}\mathcal{F}\left[e^{-\frac{\mathbf{r}\cdot[\mathbb{1}-\varepsilon\bar{\mathcal{Q}}(\varphi,\varphi')]\cdot\mathbf{r}}{2[1-\varepsilon^2\cos^2(\varphi-\varphi')]\left(l_\parallel^2+l_\perp^2\right)}}\right]. \tag{9b}$$

In order to keep the notation compact, we abbreviate the argument of the function $\mathcal{F}$ by

$$\xi = e^{-\frac{\mathbf{r}\cdot[\mathbb{1}-\varepsilon\bar{\mathcal{Q}}(\varphi,\varphi')]\cdot\mathbf{r}}{2[1-\varepsilon^2\cos^2(\varphi-\varphi')]\left(l_\parallel^2+l_\perp^2\right)}}. \tag{10}$$

Applying the chain rule, we thus obtain the intermediate result

$$\mathbf{f}_2(\mathbf{r},\varphi,\varphi') = \kappa\xi\frac{d\mathcal{F}[\xi]}{d\xi}\nabla_{\mathbf{r}}\left[\frac{\mathbf{r}\cdot\left[\mathbb{1}-\varepsilon\bar{\mathcal{Q}}(\varphi,\varphi')\right]\cdot\mathbf{r}}{2[1-\varepsilon^2\cos^2(\varphi-\varphi')](l_\parallel^2+l_\perp^2)}\right], \tag{11a}$$

$$m_2(\mathbf{r},\varphi,\varphi') = \kappa\xi\frac{d\mathcal{F}[\xi]}{d\xi}\partial_{\varphi}\left[\frac{\mathbf{r}\cdot\left[\mathbb{1}-\varepsilon\bar{\mathcal{Q}}(\varphi,\varphi')\right]\cdot\mathbf{r}}{2[1-\varepsilon^2\cos^2(\varphi-\varphi')](l_\parallel^2+l_\perp^2)}\right]. \tag{11b}$$

Performing the remaining differentiation eventually yields the expressions for force and torque. For the force, the differentiation is trivial:

$$\nabla_{\mathbf{r}}\Big\{\mathbf{r}\cdot\left[\mathbb{1}-\varepsilon\bar{\mathcal{Q}}(\varphi,\varphi')\right]\cdot\mathbf{r}\Big\} = 2\left[\mathbb{1}-\varepsilon\bar{\mathcal{Q}}(\varphi,\varphi')\right]\cdot\mathbf{r}. \tag{12}$$

Accordingly, the pairwise force exerted by one rod on another is thus determined by

$$\mathbf{f}_2(\mathbf{r},\varphi,\varphi') = \mathcal{M}_\varepsilon(\mathbf{r},\varphi,\varphi')\cdot\mathbf{r} = \mathcal{S}(\mathbf{r};\varphi,\varphi')\left[\mathbb{1}-\varepsilon\bar{\mathcal{Q}}(\varphi,\varphi')\right]\cdot\mathbf{r}, \tag{13}$$

where the kernel

$$\mathcal{S}(\mathbf{r};\varphi,\varphi') = \frac{\kappa}{l_\parallel^2+l_\perp^2}\cdot\frac{1}{1-\varepsilon^2\cos^2(\varphi-\varphi')}\cdot\left[\xi\cdot\frac{d\mathcal{F}[\xi]}{d\xi}\right] \tag{14}$$

was abbreviated for convenience. For the torque, the differentiation of the nominator in Supplementary Eq. (11b) yields the collision avoidance term, whereas the nematic alignment follows from the differentiation of the denominator:

$$m_2(\mathbf{r},\varphi,\varphi') = \frac{\mathcal{S}(\mathbf{r};\varphi,\varphi')}{2}\left\{\varepsilon|\mathbf{r}|^2\sin\left[2(\varphi-\arg(\mathbf{r}))\right]+\varepsilon^2\,\frac{\mathbf{r}\cdot\left[\mathbb{1}-\varepsilon\bar{\mathcal{Q}}(\varphi,\varphi')\right]\cdot\mathbf{r}}{1-\varepsilon^2\cos^2(\varphi-\varphi')}\,\sin\left[2(\varphi'-\varphi)\right]\right\}. \tag{15}$$

Note that in the limit $\varepsilon = 0$, the torque vanishes identically and the force reduces to an isotropic central body force.

Spurious torques in the model $u_2 = \tilde{\kappa}\mathcal{I}$. We eventually give the expressions for the torque for a model where the binary interaction energy is directly proportional to the overlap, i.e.

$$u_2(\mathbf{r};\varphi,\varphi') = \tilde{\kappa}\mathcal{I}_0(\varphi,\varphi')\,e^{-\frac{\mathbf{r}\cdot\left[\mathbb{1}-\varepsilon\bar{\mathcal{Q}}(\varphi,\varphi')\right]\cdot\mathbf{r}}{2\left[1-\varepsilon^2\cos^2(\varphi-\varphi')\right]\left(l_\parallel^2+l_\perp^2\right)}}. \tag{16}$$

The derivation is technically identical to the one of the previous paragraph. The binary torque in this model, which is obtained from $m_2(\mathbf{r},\varphi,\varphi') = -\partial_\varphi u_2(\mathbf{r};\varphi,\varphi')$, reads

$$m_2(\mathbf{r},\varphi,\varphi') = \frac{\tilde{\mathcal{S}}(\mathbf{r},\varphi,\varphi')}{2}\left\{\varepsilon|\mathbf{r}|^2\sin\left[2(\varphi-\arg(\mathbf{r}))\right]+\varepsilon^2\left[\frac{\mathbf{r}\cdot\left[\mathbb{1}-\varepsilon\bar{\mathcal{Q}}(\varphi,\varphi')\right]\cdot\mathbf{r}}{1-\varepsilon^2\cos^2(\varphi-\varphi')}-\left(l_\parallel^2+l_\perp^2\right)\right]\sin\left[2(\varphi'-\varphi)\right]\right\} \tag{17}$$

with the prefactor

$$\tilde{\mathcal{S}}(\mathbf{r},\varphi,\varphi') = \frac{u_2(\mathbf{r};\varphi,\varphi')}{\left(l_\parallel^2+l_\perp^2\right)\left[1-\varepsilon^2\cos^2\left(\varphi-\varphi'\right)\right]}. \tag{18}$$

These equations are the analogues to Supplementary Eqs. (14)-(15). The structural similarity of the corresponding terms is obvious. However, the prefactor in front of the nematic alignment term can turn negative, signaling the emergence of perpendicular alignment for

$$\frac{\mathbf{r}\cdot\left[\mathbb{1}-\varepsilon\bar{\mathcal{Q}}(\varphi,\varphi')\right]\cdot\mathbf{r}}{\left(l_\parallel^2+l_\perp^2\right)\left[1-\varepsilon^2\cos^2(\varphi-\varphi')\right]} < 1. \tag{19}$$

This inequality is never true for large inter-particle distances. However, if the particles come close, the nematic alignment term changes sign. This reflects the emergence of spurious torques which were qualitatively discussed above along with Supplementary Fig. 1. As long as large overlaps (small inter-particle distances) cannot occur due to high repulsion or weak self-propulsion, spurious torques are irrelevant. However, qualitative differences are expected if particles can cross each other, e.g. because there is a high level of fluctuations, a high level of activity or the particle density is very large. If, on the other hand, the repulsion strength is very high, those configuration will never occur and both models become equivalent.

SUPPLEMENTARY NOTE 2: ADDITIONAL LANGEVIN SIMULATIONS

Phase diagrams – identification of motility-induced phase separation. Here, we provide additional quantitative results of numerical Langevin simulations that support the main findings reported in the main text. At first, we present phase diagrams for different aspect ratios to identify regions in parameter space where motility-induced phase separation emerges. We focus on the plane of density ρ_0 and activity/persistence, quantified here by the Péclet number defined as $v_0/(\ell D_\varphi)$, with the characteristic length $\ell = \sqrt{l_\parallel l_\parallel}$; note, however, that we fix $l_\parallel l_\perp = 1$ in all simulations. Different measures to identify MIPS were proposed in literature, such as the structure of the local density distribution (unimodal vs. bimodal) or

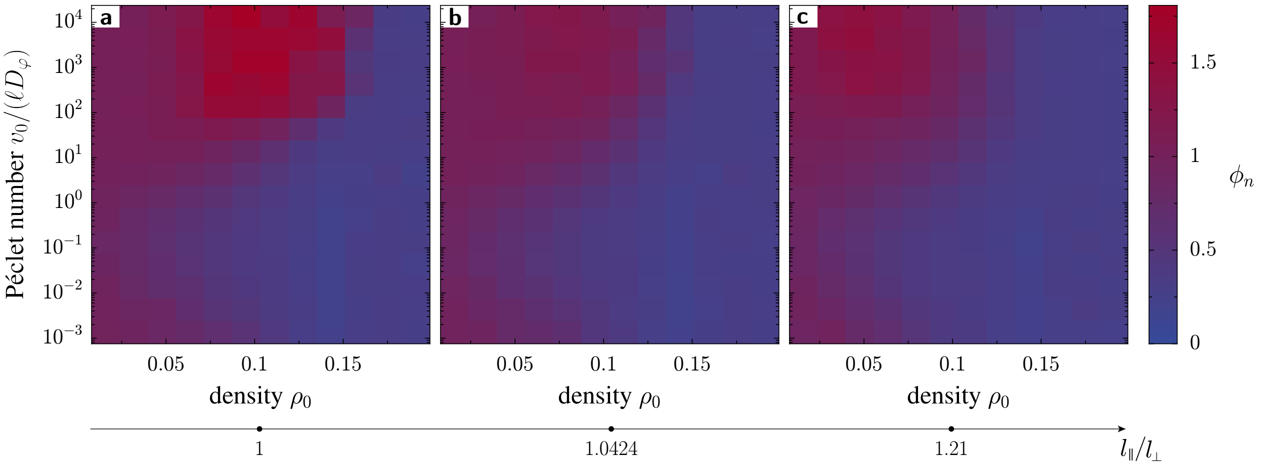

Supplementary Figure 2 | Phase diagram in the density-activity plane for three different anisotropies. Color-coded is the order parameter ϕ_n for the level of density fluctuations, calculated by sub-dividing the system into boxes of length l_b and calculating the standard deviation of the local particle number in comparison to the expected value for a Poissonian point pattern (confer the respective paragraph in the text for additional technical details). Panel **a** corresponds to self-propelled spheres ($\varepsilon = 0$, $l_\parallel/l_\perp = 1$); panels **b** and **c** show the same quantity for increasing anisotropies: $l_\parallel/l_\perp = 1.0424$ and $l_\parallel/l_\perp = 1.21$, respectively. The level of density fluctuations is lower for slightly anisotropic rods. Representative snapshots are discussed in the context of Fig. 2 in the main text of the manuscript; see the caption of Fig. 2 for simulation parameters.

cluster size distributions. In order to identify MIPS beyond the visual inspection of snapshots of simulations, we measured two order parameters that should capture central properties of MIPS. Motility-induced phase separation, as it is commonly described for self-propelled discs, is a phenomenon where a system spontaneously phase separates into a dense phase with local hexatic order and a low density, disordered gas phase. In short, two structural properties are relevant: (i) density fluctuations and (ii) the level of hexatic order.

For the measurement of density fluctuations, the system is subdivided into N_b boxes of edge length $l_b = 10$ and the number of particles n_j is counted in each box j. The expected number of particles in a box is $\rho_0 l_b^2$ for the particle density ρ_0. We calculate the standard deviation of the local particle number std(n) and construct an order parameter ϕ_n by dividing by the standard deviation for a Poissonian point pattern that is given by $\sqrt{Np(1-p)}$ with $p = l_b^2/L^2$. Accordingly, this parameter equals one for a Poissonian point pattern and it is significantly larger than one for a strongly inhomogeneous distribution of particles; values smaller than one indicate regular arrangements of particles (e.g. a lattice). The level of density fluctuations in the density-activity plane is shown in Supplementary Fig. 2 for three different aspect ratios.

The measurement of the level of local hexatic order is based on a Voronoi tesselation [3]. The Voronoi neighbors Ω_j for each particle j are identified first. Then, the complex order parameter

$$\psi_j = \frac{1}{|\Omega_j|} \sum_{k\in\Omega_j} e^{6i\alpha_{kj}} \tag{20}$$

is calculated for each particle, where α_{kj} is the polar angle of the displacement vector $\mathbf{r}_k - \mathbf{r}_j$. The level of local hexatic order is given by $|\psi_j|$. The breakdown of MIPS for anisotropic particles as shown in Fig. 2 of the main text is accompanied by the breakdown of local hexatic order: Supplementary Fig. 3 shows the same states like Fig. 2a,b, but the color coding corresponds to the local hexatic order parameter. In addition, we measured the distribution of the local particle number that reveals a bimodal structure for self-propelled spheres, whereas it is unimodal for rods.

The average level of local hexatic order is quantified by averaging ψ_j over particles all particles:

$$\phi_{h_6} = \frac{1}{N} \sum_{j=1}^{N} |\psi_j| \,. \tag{21}$$

These considerations eventually allow to construct an order parameter for the identification of motility-induced phase separation, that is characterized by the simultaneous presence of high density fluctuations and the emergence of local hexatic order, via the product of ϕ_n and ϕ_{h_6}. Corresponding phase diagrams are shown in Supplementary Fig. 4 for different aspect ratios in the density-activity plane. These quantitative phase diagrams reveal that motility-induced phase separation does only emerge for self-propelled spheres, whereas the order parameter is significantly reduced for self-propelled rods. A mechanistic explanation why MIPS breaks down is discussed in the main text along with analytical arguments.

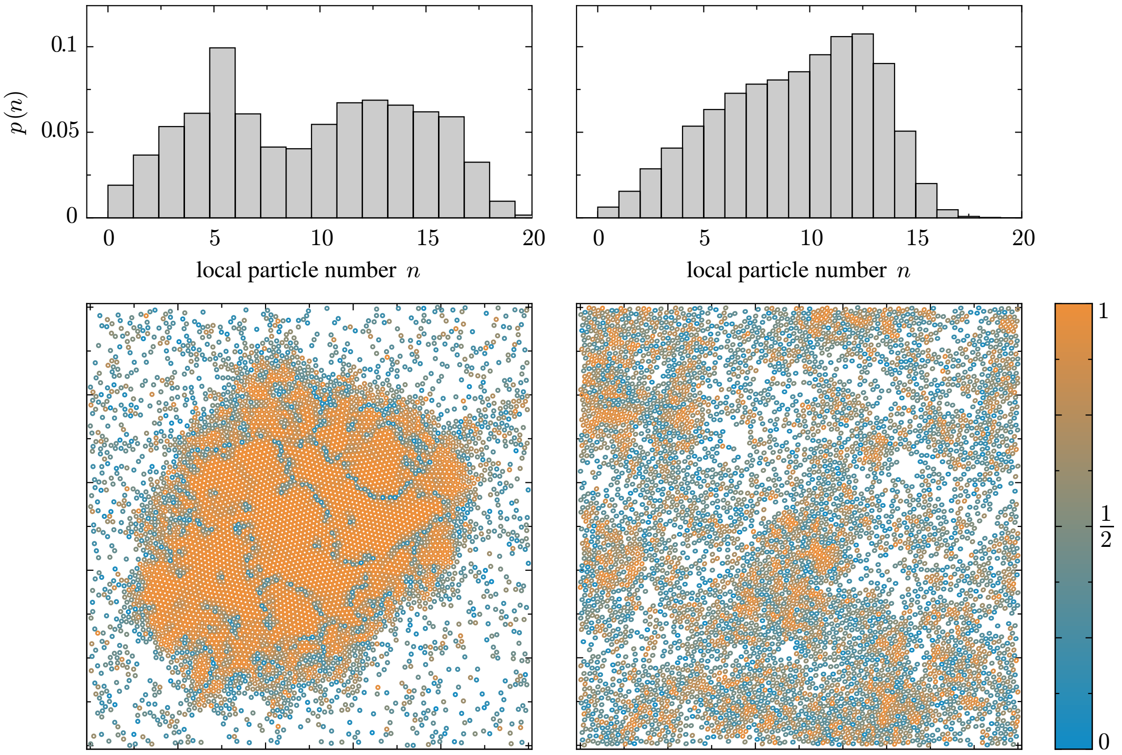

Supplementary Figure 3 | Motility-induced phase separation of self-propelled discs and its break-down for self-propelled rods. The same states like in the first two panels of Fig. 2 in the main text are shown, however, the color coding corresponds to the absolute value of the local hexatic order parameter $|\psi_j|$, calculated for Voronoi neighbors [3]. Left: in the case of spheres, the system phase separates into a hexatic aggregate and a disordered gas; right: for weak anisotropies of rods, the density fluctuations and the level of hexatic order are reduced due to anisotropic, active stresses. The panels on the top show the corresponding distributions of the local number of neighbors (counted by sub-dividing the system into boxes as explained above) which is bimodal in the phase-separated state and unimodal otherwise. For simulation parameters, see Figs. 2(a,b).

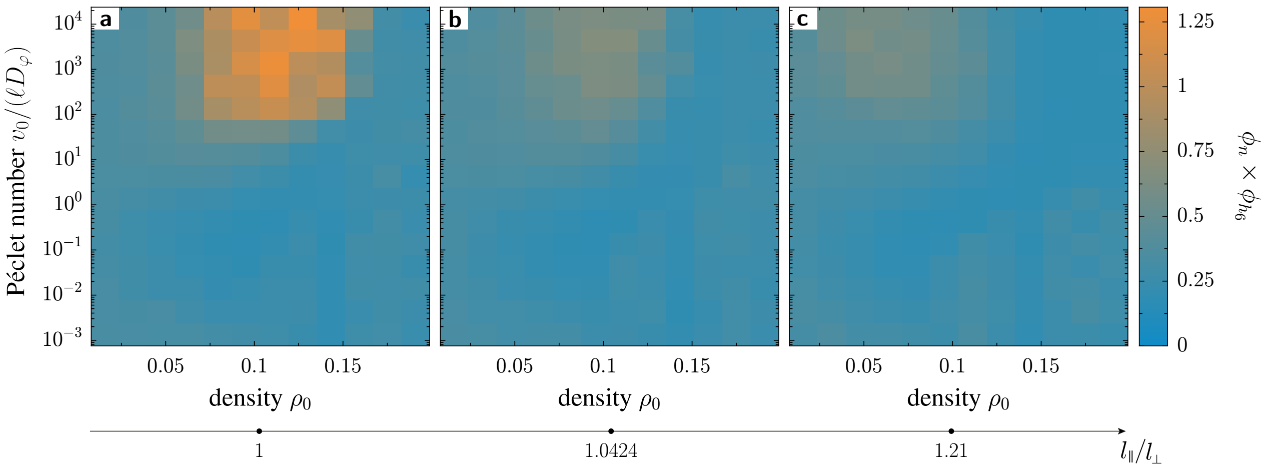

Supplementary Figure 4 | Phase diagram in the density-activity plane for three different anisotropies (cf. Supplementary Fig. 2). Color-coded is the order parameter $\phi_n \times \phi_{h_6}$, that is the product of the order parameter for density fluctuations and the average level of local hexatic order. The characteristic parabolic parameter regime where MIPS emerges is observed for self-propelled discs (panel **a**), whereas it is not observed for self-propelled rods (panels **b** and **c**).

Particle transport. To underline the different structural properties of the states that are illustrated in Fig. 2, we quantified the transport properties of particles by measuring the mean-squared displacement

$$\left\langle |\Delta\mathbf{r}|^2 \right\rangle = \frac{1}{N} \sum_j \left| \mathbf{r}_j(t) - \mathbf{r}_j(t_0) \right|^2 \tag{22}$$

as a function of time $\Delta t = t - t_0$, graphically represented in Supplementary Fig. 5. As initial conditions, we chose the states that are shown in Fig. 2.

As commented in the context of Eq. (29) in the main text, one could, in principle, alternatively use the expressions for effective transport coefficients to quantify numerically the transport properties of the system, e.g. the collective diffusion coefficient Γ of the density field [4]. Note, however, that the expression for the collective diffusion coefficient was derived in the context of the linear stability analysis of the spatially homogeneous, isotropic state. This requires weak density fluctuations as well as the absence of both, local and global order since the local polar order parameter was adiabatically eliminated in this derivation.

The resulting expression for Γ is therefore, strictly speaking, only applicable in the disordered phase; it can, moreover, be used to detect the linear threshold to the emergence of MIPS [4]. However, as soon as local order emerges, the expression for Γ cannot be interpreted as the collective diffusion coefficient any longer, as the assumptions which led to its derivation are not longer justifiable. Thus, the applicability of this measure is limited to a small range of aspect ratios only (disc-like particles). For this reason, we decided to quantify the transport properties of the system via the mean-squared displacement at the single-particle level. For disordered states, where the collective diffusion coefficient can be measured numerically such as in the case of self-propelled discs, its measurement and the transport characteristics derived from the single-particle mean-squared displacement are expected to yield identical predictions.

The numerical study reveals a key difference between self-propelled discs and self-propelled rods: whereas the aggregates that are formed via MIPS rarely move – they are globally disordered and move diffusively with a diffusion coefficient that decreases as the size of the aggregate grows – polar clusters formed by self-propelled rods are highly dynamic and move ballistically. This is because polar ordering is inherently related to convective mass transport. In fact, polar clusters are becoming more persistent, i.e. ballistic, as they grow in size as rotational diffusion is suppressed for particles inside clusters. Technically speaking, MIPS is based on diffusion mediated transport – drops grow in size by exchanging particles/clusters via a diffusion process – while, in sharp contrast, the cluster dynamics of elongated active particles results from the interaction of polar cluster moving ballistically, with a persistence that increases with cluster size [5, 6].

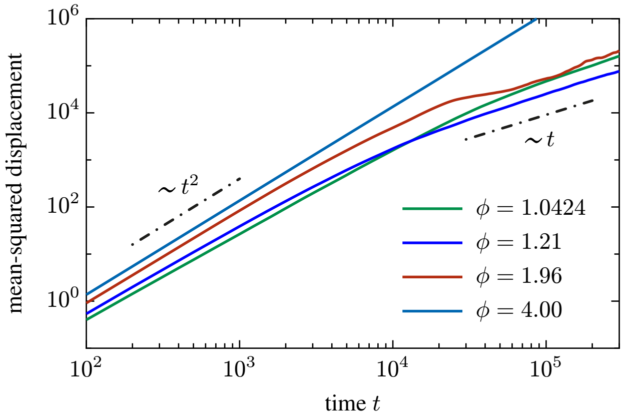

Supplementary Figure 5 | Mean-squared displacement as a measure for collective transport properties. The simulation parameters correspond to the snapshots shown in Fig. 2 of the main text. Notably, the effective speed and persistence of the collective dynamics depends on the aspect ratio ϕ of particles. In particular, elongated self-propelled rods move highly persistent and ballistically.

Positional ordering and substructure of polar bands. In the main text, we highlighted that the polar band formed by self-propelled rods for high aspect ratios has a nontrivial substructure (cf. Fig. 2e): particles that are aligned tend to move next to each other, thereby forming smectic layers. To explain this type of ordering, we point out the fixed points of Eq. (3) that contains both, an alignment-like torque as well as a torque that couples the orientation of a rod to the relative position of its interaction partner. The latter interaction mechanism favors configurations where two rods arrange such that their body axis and the vector that connects the two centers of mass are perpendicular to each other. This torque is responsible for the formation of a non-trivial substructure which, in contrast, cannot be observed in Vicsek-like alignment models for self-propelled rods [7] where this type of coupling is absent.

We constructed two order parameters to quantify the formation of these substructures in more detail. Supplementary Fig. 6 shows the same snapshot as Fig. 2e of the main text, but the color-coding corresponds to different order parameters

that quantify the local structural properties as follows. Both order parameters are based on a Voronoi tesselation, i.e. local quantities are associated to particles by summing over Voronoi neighbors. Our analysis reveals that the band is composed of patches within which they form quadratic, anisotropic lattices that are revealed by the order parameter

$$\Lambda_1^{(j)} = \frac{1}{|\Omega_j|} \sum_{k\in\Omega_j} e^{4i\alpha_{kj}}. \tag{23}$$

This definition is structurally related to the local hexatic order parameter discussed above. It is shown in Supplementary Fig. 6a.

In line with our argument above that the torque $\dot{\varphi}_i \propto \sin[2(\varphi_i - \alpha_{ij})]$ favors perpendicular alignment of the relative distance vector connecting the centers of mass of two rods and the direction of motion of a rod, one may define an effective interaction energy proportional to $\cos^2(\varphi_i - \alpha_{ij})$. We associate to each particle the local average of this quantity,

$$\Lambda_2^{(j)} = \frac{1}{|\Omega_j|} \sum_{k\in\Omega_j} \cos^2(\varphi_j - \alpha_{kj}), \tag{24}$$

as shown in Supplementary Fig. 6b that reveals a high level of positional ordering in almost all parts of the band.

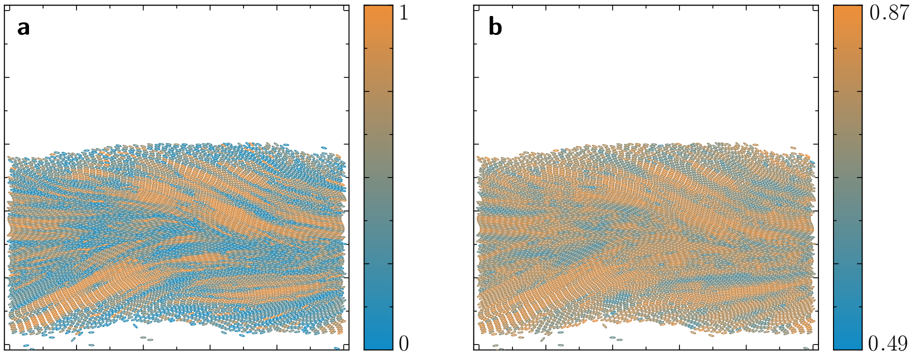

Supplementary Figure 6 | Characterization of the local structure of the polar band formed by self-propelled rods. The snapshots correspond to the state that is shown in Fig. 2e. Here, color-coding represent different order parameters to quantify the structural properties of the band, quantified by the order parameters Λ_1 in panel **a** and Λ_2 in panel **b**, cf. Supplementary Eqs. (23) and (24), respectively.

SUPPLEMENTARY NOTE 3: KINETIC THEORY

Fokker-Planck dynamics of the one-particle density. In this appendix, we summarize some technical details on the kinetic theory discussed in the main text. The central observable linking particle-based descriptions and a field theoretical treatment is the one-particle density distribution

$$P(\mathbf{r},\varphi,t) = \Big\langle \textstyle\sum_{j=1}^{N} \delta\big(\mathbf{r}-\mathbf{r}_j(t)\big)\delta\big(\varphi-\varphi_j(t)\big)\Big\rangle, \tag{25}$$

which determines the density of particles at a particular reference point in phase space $\{\mathbf{r},\varphi\}$. Its dynamics

$$\partial_t P = -\nabla\cdot\Big[\big(v_0\mathbf{e}_{\parallel}[\varphi] + \hat{\boldsymbol{\mu}}[\varphi]\cdot\mathbf{F}\big)P\Big] + \nabla\cdot\Big[\mathcal{D}[\varphi]\cdot\nabla P\Big] - \partial_\varphi\Big[\mu_\varphi M P\Big] + D_\varphi\partial_\varphi^2 P \tag{26}$$

can be derived from the N-particle Fokker-Planck equation [8] corresponding to the particle-based rod dynamics. In general, however, the dynamics of the one-particle density is not closed but it depends on the pair correlation function g_2 via the force and torque functionals:

$$\mathbf{F}(\mathbf{r},\varphi,t) = \int d^2r' d\varphi'\, \mathbf{f}_2(\mathbf{r}-\mathbf{r}',\varphi,\varphi')\, P(\mathbf{r}',\varphi',t)\, g_2(\mathbf{r},\mathbf{r}';\varphi,\varphi',t), \tag{27a}$$

$$M(\mathbf{r},\varphi,t) = \int d^2r' d\varphi'\, m_2(\mathbf{r}-\mathbf{r}',\varphi,\varphi')\, P(\mathbf{r}',\varphi',t)\, g_2(\mathbf{r},\mathbf{r}';\varphi,\varphi',t). \tag{27b}$$

Thus, an appropriate closure scheme has to be found in order to address physical questions within kinetic theory. The explanation of the phenomena addressed in the main text – active phase separation and its breakdown as well as the emergence of polar order from nematic interactions – cannot be explained on the mean-field level ($g_2 \approx 1$) but requires the consideration of inter-particle correlations as they are caused by the kinetics of collisions.

The spherical limit approximation – from particles to fields. In order to obtain the field theoretical equivalents of particle-based forces and torques, the kernels $\mathbf{f}_2(\mathbf{r},\varphi,\varphi')$ and $m_2(\mathbf{r},\varphi,\varphi')$, cf. Supplementary Eqs. (13) and (15), have to be inserted into the corresponding interaction integrals above. In the spherical limit approximation, these expressions are substantially simplified:

$$\mathbf{f}_2(\mathbf{r},\varphi,\varphi') \simeq \kappa\left[\xi\cdot\frac{d\mathcal{F}[\xi]}{d\xi}\right]\frac{\mathbf{r}}{2l^2}+\mathcal{O}(\varepsilon), \tag{28a}$$

$$m_2(\mathbf{r},\varphi,\varphi') \simeq \kappa\varepsilon\left[\xi\cdot\frac{d\mathcal{F}[\xi]}{d\xi}\right]\frac{|\mathbf{r}|^2}{4l^2}\sin\big[2(\varphi-\arg(\mathbf{r}))\big]+\mathcal{O}\big(\varepsilon^2\big). \tag{28b}$$

Several further simplifications are helpful. First, the one-particle density in the interaction integrals is expanded into a Taylor series in spatial gradients. Furthermore, an ansatz for g_2 has to be provided. We neglected its explicit temporal dependence and, further, reduced the number of variables of the pair-distribution using global symmetries, namely spatial homogeneity and rotational invariance:

$$g_2(\mathbf{r},\mathbf{r}';\varphi,\varphi',t) \simeq \tilde{g}_2(|\mathbf{r}-\mathbf{r}'|,\arg(\mathbf{r}'-\mathbf{r})-\varphi,\varphi'-\varphi). \tag{29}$$

In the main text, we reported that the most relevant contribution to the two-particle distribution function is the kinetic enhancement of the particle density in front of a focal particle with respect to its direction of motion due to self-propulsion: as particles move actively in a semi-dilute environment, they tend to collide with others, and consequently the probability to find a particle in front is higher than in the back. We take this effects into account considering the corresponding, first Fourier component of g_2:

$$\tilde{g}_2(|\mathbf{r}-\mathbf{r}'|,\arg(\mathbf{r}'-\mathbf{r})-\varphi,\varphi'-\varphi) \simeq \tilde{g}_2^{(0)}(|\mathbf{r}-\mathbf{r}'|)+\tilde{g}_2^{(1)}(|\mathbf{r}-\mathbf{r}'|)\cos\big[\arg(\mathbf{r}'-\mathbf{r})-\varphi\big]. \tag{30}$$

Using these approximations, we obtain the substantially simplified force and torque functionals

$$\mathbf{F} \simeq -\zeta_0\kappa\mathbf{e}_{\parallel}[\varphi]\rho, \tag{31a}$$

$$M \simeq -\varepsilon\zeta_1\kappa\mathbf{e}_{\parallel}[\varphi]\wedge\nabla\rho = -\varepsilon\zeta_1(\cos\varphi\partial_y-\sin\varphi\partial_x)\rho \tag{31b}$$

with the density field

$$\rho(\mathbf{r},t) = \int_{-\pi}^{\pi} d\varphi\, P(\mathbf{r},\varphi,t) \tag{32}$$

and the transport coefficients

$$\zeta_0 = -\pi\int_0^\infty dr\, r\tilde{g}_2^{(1)}(r)\,\frac{d\mathcal{F}\Big[e^{-r^2/(4l^2)}\Big]}{dr}\,, \tag{33a}$$

$$\zeta_1 = -\frac{\pi}{4}\int_0^\infty dr\, r\tilde{g}_2^{(1)}(r)\,\frac{d\mathcal{F}\Big[e^{-r^2/(4l^2)}\Big]}{dr}\cdot\Big(\frac{r}{l}\Big)^2. \tag{33b}$$

The coefficients ζ_i are positive if the density is enhanced in front of a particle with respect to its direction of motion as argued in the main text.

Derivation of Eqs. (15,16). In the main text, the transport coefficients σ_p and σ_n which determine the symmetry breaking of the polar and nematic order parameter at the local level, defined by

$$\dot{\mathbf{p}} = \sigma_p\mathbf{p}+\mathcal{O}(\nabla), \tag{34a}$$

$$\dot{\mathfrak{Q}} = \sigma_n\mathfrak{Q}+\mathcal{O}(\nabla), \tag{34b}$$

are discussed [Eqs. (15,16) in the main text]. Details on their derivation are summarized in this appendix.

The derivation follows essentially the steps outlined in the corresponding Methods section: the temporal dynamics of the one-particle density distribution $P(\mathbf{r},\varphi,t)$ given by Supplementary Eq. (26) is successively inserted into the definition of the corresponding order parameters:

$$\partial_t\mathbf{p}(\mathbf{r},t) = \int_{-\pi}^{\pi} d\varphi\,\mathbf{e}_{\parallel}[\varphi]\,\partial_t P(\mathbf{r},\varphi,t) = \int_{-\pi}^{\pi} d\varphi\begin{pmatrix}\cos\varphi\\ \sin\varphi\end{pmatrix}\partial_t P(\mathbf{r},\varphi,t), \tag{35a}$$

$$\partial_t\mathfrak{Q}(\mathbf{r},t) = \int_{-\pi}^{\pi} d\varphi\,\mathcal{Q}[\varphi]\,\partial_t P(\mathbf{r},\varphi,t) = \int_{-\pi}^{\pi} d\varphi\begin{pmatrix}\cos(2\varphi) & \sin(2\varphi)\\ \sin(2\varphi) & -\cos(2\varphi)\end{pmatrix}\partial_t P(\mathbf{r},\varphi,t). \tag{35b}$$

In two dimensions, there is, however, a more elegant and direct way to calculate both transport coefficients at once via Fourier transform with respect to the angular variable φ. For this purpose, we first introduce the angular Fourier modes of the one-particle density distribution as follows:

$$f_n(\mathbf{r},t) = \int_0^{2\pi} d\varphi\, e^{in\varphi} P(\mathbf{r},\varphi,t), \quad P(\mathbf{r},\varphi,t) = \frac{1}{2\pi}\sum_n e^{-in\varphi} f_n(\mathbf{r},t). \tag{36}$$

Order parameters and angular Fourier modes are directly related via

$$\rho(\mathbf{r},t) = f_0(\mathbf{r},t), \quad \mathbf{p}(\mathbf{r},t) = \begin{pmatrix} \Re[f_1(\mathbf{r},t)] \\ \Im[f_1(\mathbf{r},t)] \end{pmatrix}, \quad \mathfrak{Q}(\mathbf{r},t) = \begin{pmatrix} \Re[f_2(\mathbf{r},t)] & \Im[f_2(\mathbf{r},t)] \\ \Im[f_2(\mathbf{r},t)] & -\Re[f_2(\mathbf{r},t)] \end{pmatrix}. \tag{37}$$

Since we are solely interested in the transport coefficients at the local level, gradient terms are neglected in the following discussion. Accordingly, only the terms involving torque and angular diffusion in the Fokker-Planck equation [Supplementary Eq. (26)] are relevant. Their Fourier transform reads

$$\partial_t f_n = \frac{in\mu_\varphi}{2\pi}\sum_m M_m f_{n-m} - n^2 D_\varphi f_n + \mathcal{O}(\nabla), \tag{38}$$

where

$$M_n(\mathbf{r},t) = \int_0^{2\pi} d\varphi\, e^{in\varphi} M(\mathbf{r},\varphi,t) \tag{39}$$

denotes the angular Fourier modes of the torque functional [Supplementary Eq. (27b)] at the coarse-grained level. As a next step, we use symmetry properties of the model to simplify the torque functional

$$M(\mathbf{r},\varphi,t) = \int d^2r' d\varphi'\, P(\mathbf{r}',\varphi',t) m_2(\mathbf{r}-\mathbf{r}',\varphi,\varphi') g_2(\mathbf{r},\mathbf{r}';\varphi,\varphi',t) \tag{40}$$

and the subsequent calculation of Fourier modes. At first, the one particle distribution function $P(\mathbf{r}',\varphi,t)$ can be expanded in Taylor series in $\mathbf{r}'$ around the point $\mathbf{r}$ because we focus on the local level and, moreover, interactions are local as they are based on the contact of two particles:

$$M(\mathbf{r},\varphi,t) \approx \int d^2r' d\varphi'\, P(\mathbf{r},\varphi',t) m_2(\mathbf{r}-\mathbf{r}',\varphi,\varphi')\, g_2(\mathbf{r},\mathbf{r}';\varphi,\varphi',t) + \mathcal{O}(\nabla) \tag{41a}$$

$$= \frac{1}{2\pi}\sum_n f_n(\mathbf{r},t) \int d^2r' d\varphi'\, e^{-in\varphi'} m_2(\mathbf{r}-\mathbf{r}',\varphi,\varphi') g_2(\mathbf{r},\mathbf{r}';\varphi,\varphi',t) + \mathcal{O}(\nabla). \tag{41b}$$

In parallel to the discussion in the main text, we introduce an effective mean-field torque $\tilde{m}_2 = m_2 g_2$ which combines the effects of the actual interaction m_2 and the collision kinetics, described by the pair correlation function g_2. We refer to it as an effective *mean-field* model because structurally identical equations were obtained if $\tilde{m}_2$ was the actual microscopic interaction in a system that is studied within mean-field approximation, where correlations are neglected ($g_2 \approx 1$). Note, however, that in the present context correlations are included into the theory as we do not specify or presuppose a particular structure of the pair-correlation function.

By virtue of rotational and translational invariance of the model, the number of variables of $\tilde{m}_2$ can be reduced via $\tilde{m}_2 = \tilde{m}_2(|\mathbf{r}-\mathbf{r}'|, \alpha-\varphi, \varphi'-\varphi)$, where $\alpha = \arg(\mathbf{r}-\mathbf{r})$. In words, this reflects that the interaction does only depend on the relative position and relative orientation of one particle with respect to another one. This can be used to simplify the torque functional as follows:

$$M(\mathbf{r},\varphi,t) = \frac{1}{2\pi}\sum_n f_n(\mathbf{r},t) \int_0^\infty dr'\, r' \int_0^{2\pi} d\alpha \int_0^{2\pi} d\varphi'\, e^{-in\varphi'} \tilde{m}_2(r',\alpha-\varphi,\varphi'-\varphi) + \mathcal{O}(\nabla) \tag{42a}$$

$$= \frac{1}{2\pi}\sum_n e^{-in\varphi} f_n(\mathbf{r},t) \int_0^\infty dr'\, r' \int_0^{2\pi} d\alpha \int_0^{2\pi} d\varphi'\, e^{-in\varphi'} \tilde{m}_2(r',\alpha,\varphi') + \mathcal{O}(\nabla). \tag{42b}$$

From the last line, one can directly read off the Fourier modes of the torque functional:

$$M_n(\mathbf{r},t) = f_n(\mathbf{r},t) \int_0^\infty dr'\, r' \int_0^{2\pi} d\alpha \int_0^{2\pi} d\varphi'\, e^{-in\varphi'} \tilde{m}_2(r',\alpha,\varphi') + \mathcal{O}(\nabla). \tag{43}$$

As a last step, we use the fact that the interaction is anti-symmetric with respect to reflections at the axis that is parallel to the orientation of a focal particle: $\tilde{m}_2(r, -\alpha, -\varphi') = -\tilde{m}_2(r, \alpha, \varphi')$. Accordingly, only the $\sin(n\varphi)$-terms will contribute to the integration:

$$M_n(\mathbf{r}, t) = -i f_n(\mathbf{r}, t) \int_0^\infty dr'\, r' \int_0^{2\pi} d\alpha \int_0^{2\pi} d\varphi' \, \sin(n\varphi') \tilde{m}_2(r', \alpha, \varphi') + \mathcal{O}(\nabla). \tag{44}$$

One may rewrite in this order (neglecting gradient terms) $M_n = -i\gamma_n f_n$ with the coefficients

$$\gamma_n = \int_0^\infty dr'\, r' \int_0^{2\pi} d\alpha \int_0^{2\pi} d\varphi' \, \sin(n\varphi') \tilde{m}_2(r', \alpha, \varphi'). \tag{45}$$

Note that this implies in particular $\gamma_0 = 0$.

Inserting the angular modes of the torque functional $M_n = -i\gamma_n f_n$ into the dynamics of Fourier modes f_n, cf. Supplementary Eq. (38), yields

$$\partial_t f_n = \frac{n\mu_\varphi}{2\pi} \sum_m \gamma_m f_m f_{n-m} - n^2 D_\varphi f_n + \mathcal{O}(\nabla). \tag{46}$$

The dynamics is not closed but represents an infinite hierarchy of Fourier modes. In general, a closure relation is needed which depends on the structure of state or pattern which shall be described. The closure scheme will, however, only affect nonlinear terms. The relevant terms, which determine σ_p and σ_n that were discussed in the main text, readily follow from the summand $m = n$:

$$\sigma_p = \frac{\mu_\varphi \gamma_1 \rho}{2\pi} - D_\varphi, \quad \sigma_n = \frac{\mu_\varphi \gamma_2 \rho}{\pi} - 4D_\varphi. \tag{47}$$

These expressions are the basis for the discussion in the main text.

SUPPLEMENTARY REFERENCES

[1] Buckingham, E. On physically similar systems; illustrations of the use of dimensional equations. *Phys. Rev.* **4**, 345–376 (1914).
[2] Onsager, L. The effects of shape on the interaction of colloidal particles. *Ann. N.Y. Acad. Sci.* **51**, 627–659 (1949).
[3] Klein, R. *Algorithmische Geometrie: Grundlagen, Methoden, Anwendungen* (Springer, 2005).
[4] van Damme, R., Rodenburg, J., van Roij, R. & Dijkstra, M. Interparticle torques suppress motility-induced phase separation for rodlike particles. *J. Chem. Phys.* **150**, 164501 (2019).
[5] Peruani, F. & Bär, M. A kinetic model and scaling properties of non-equilibrium clustering of self-propelled particles. *New J. Phys.* **15**, 065009 (2013).
[6] Bär, M., Großmann, R., Heidenreich, S. & Peruani, F. Self-propelled rods: Insights and perspectives for active matter. *Annu. Rev. Condens. Matter Phys.* **11**, 441–466 (2020).
[7] Ginelli, F., Peruani, F., Bär, M. & Chaté, H. Large-scale collective properties of self-propelled rods. *Phys. Rev. Lett.* **104**, 184502 (2010).
[8] Risken, H. *The Fokker-Planck equation: Methods of solution and applications* (Springer, 1996).
