# Supplementary material for: A particle-field approach bridges phase separation and collective motion in active matter
Source: Nat Commun. 2020 Oct 23;11:5365. doi: 10.1038/s41467-020-18978-5 (PMC7584633; doi:10.1038/s41467-020-18978-5)
Supplement: Supplementary file 3 — Description of Additional Supplementary Files [file 41467_2020_18978_MOESM3_ESM.pdf]

Description of Additional Supplementary Files

- Supplementary Movie 1: Illustration of MIPS formation, corresponding to Fig. 2a.
- Supplementary Movie 2: Illustration of polar domain formation, corresponding to Fig. 2d.
- Supplementary Movie 3: Illustration of polar band formation and local smectic ordering, corresponding to Fig. 2e.
- Supplementary Movie 4: Illustration of polar/nematic bistability, corresponding to Fig. 4b.
- Supplementary Movie 5: Illustration of a polar binary collision between two rods, cf. Fig. 1c; parameters from Fig. 2e.
- Supplementary Movie 6: Illustration of a polar binary collision between two rods, cf. Supplementary Movie 5, however with a different impact parameter.
- Supplementary Movie 7: Illustration of an antipolar binary collision between two rods, cf. Fig. 1d, resulting in anti-parallel alignment; parameters from Fig. 2e.
- Supplementary Movie 8: Illustration of an antipolar binary collision between two rods, cf. Supplementary Movie 7, however, with a different impact parameter, resulting in polar alignment.
